# Supplementary figures and images for: Identification of cell type specific ACE2 modifiers by CRISPR screening
Source: PLoS Pathog. 2022 Mar 1;18(3):e1010377. doi: 10.1371/journal.ppat.1010377 (PMC8929698; doi:10.1371/journal.ppat.1010377)

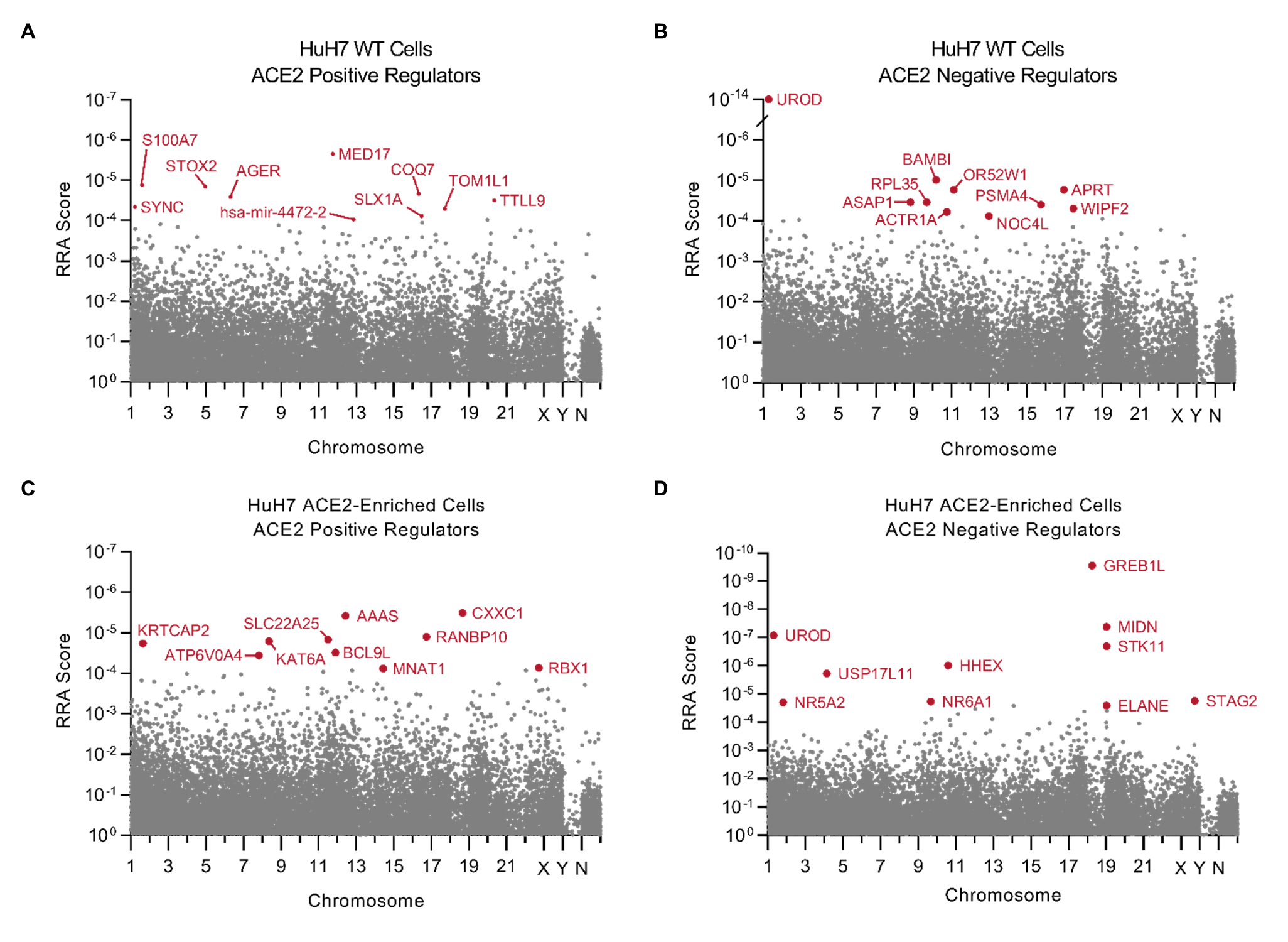

Supplement: S1 Fig — Manhattan plots demonstrating gene-level MAGeCK Robust Rank Aggregation (RRA) scores for each gene in the GeCKOv2 library plotted according to its chromosomal transcription start site. N = nontargeting controls. Plots are displayed for both positive regulation (A, C; gRNAs depleted in ACE2-positive relative to ACE2-negative populations) and negative regulation (B, D; gRNAs enriched in ACE2-positive relative to ACE2-negative populations) for independent screens of HuH7 wild-type cells (A, C) and HuH7 ACE2-enriched cells (B, D). The top 10 genes for each analysis are highlighted in red. (TIF) [file ppat.1010377.s001.tif]

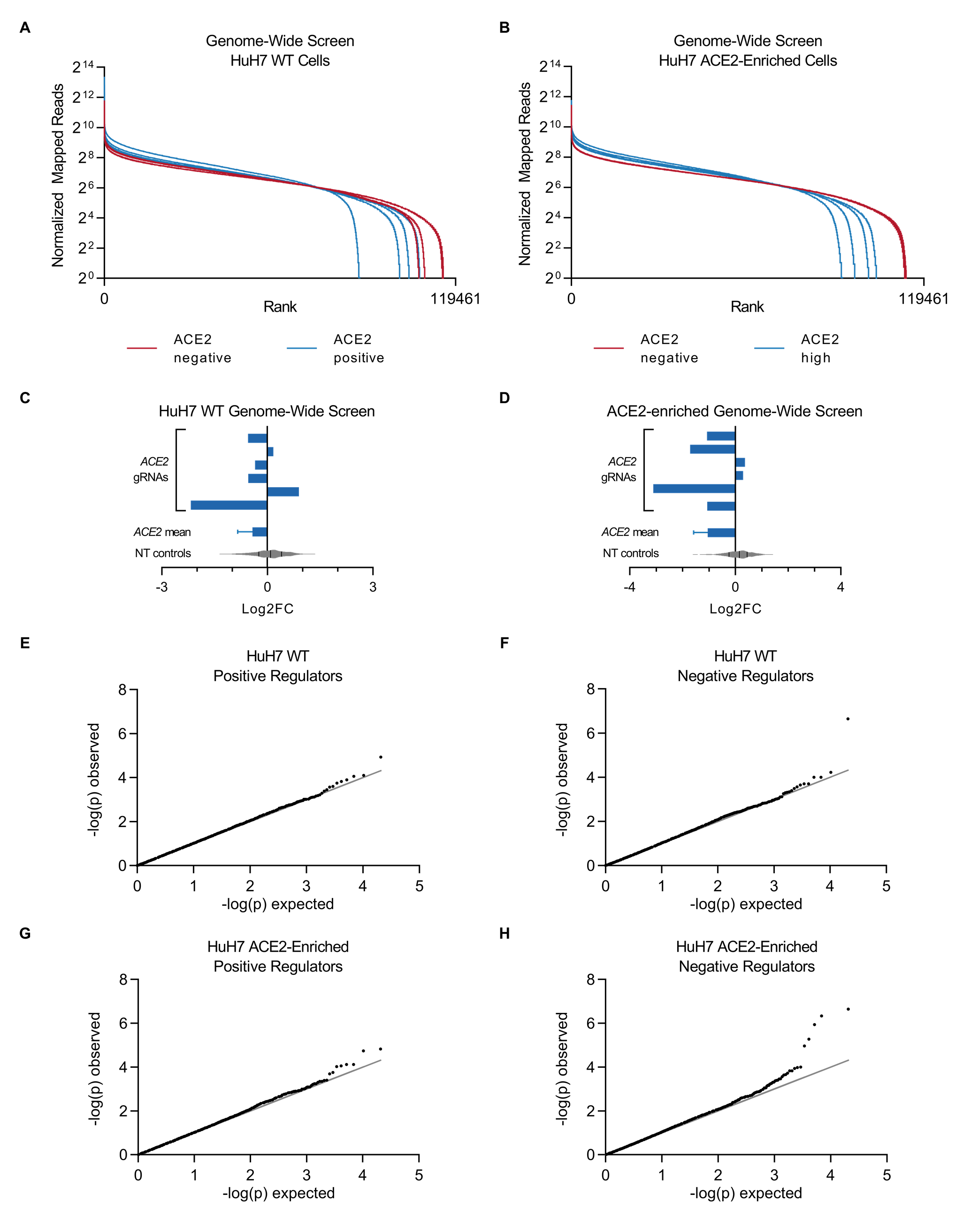

Supplement: S2 Fig — (A-B) Cumulative distribution functions of normalized read counts for each gRNA in ACE2-sorted populations in 4 independent biologic replicates of the genome-wide primary CRISPR screen of wild-type (A) and ACE2-enriched (B) HuH7 cells. (C) Mean log2 fold-change of each individual gRNA targeting ACE2, the aggregate mean of these individual ACE2-targeting gRNAs, and a violin plot of the distribution for all 1000 control nontargeting gRNAS (lines indicate quartiles) in the genome-wide ACE2 CRISPR screens of wild-type (C) and ACE2-enriched (D) HuH7 cells. (E-H) Q-Q plots of observed versus expected–log(p) for positive (E,G) or negative (F, H) regulation of ACE2 surface abundance for every gene in the primary genome-wide CRISPR screen of HuH7 wild-type (E-F) or ACE2-enriched (G-H) cells. Observed p-values were calculated by MAGeCK gene-level analysis. (TIF) [file ppat.1010377.s002.tif]

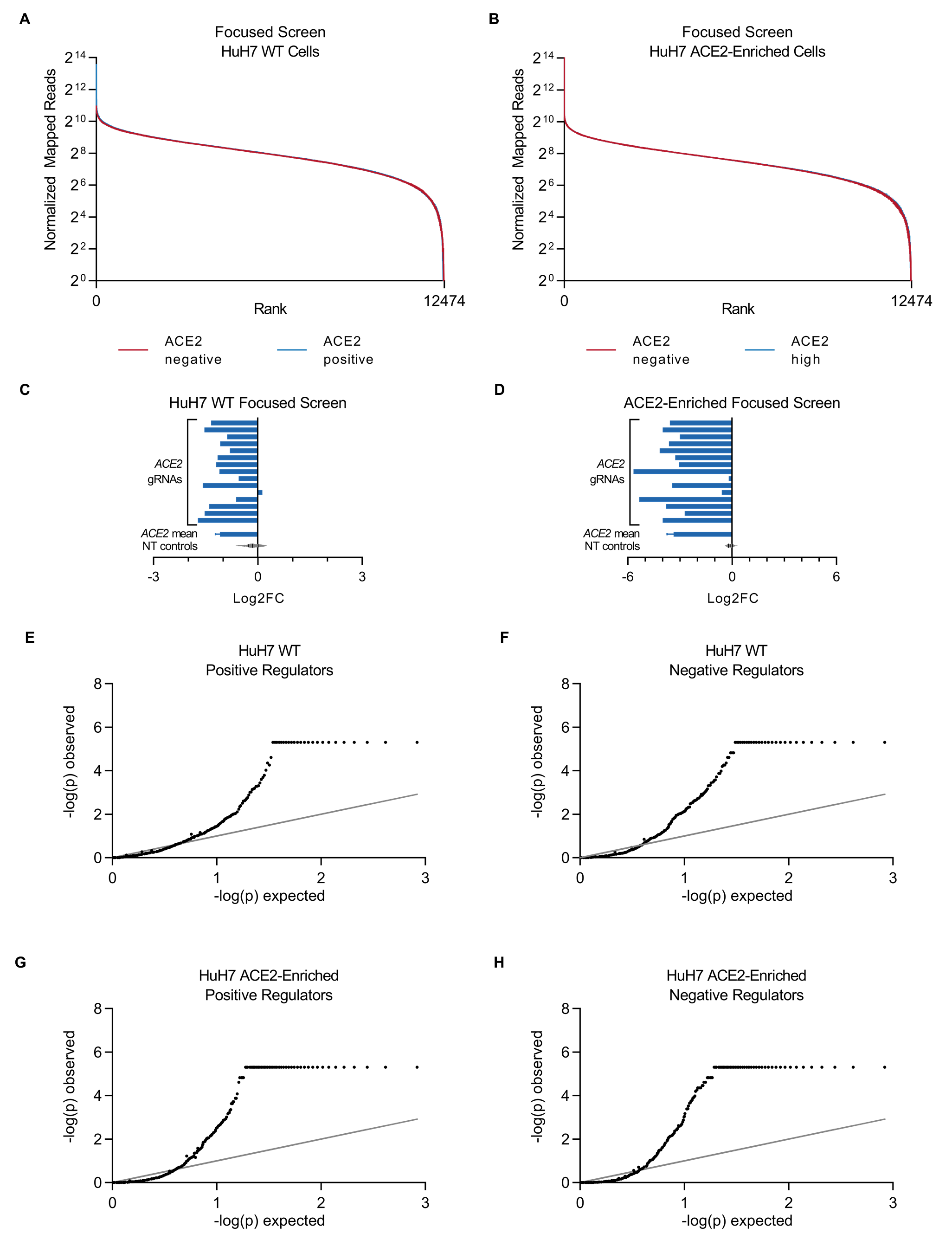

Supplement: S3 Fig — (A-B) Cumulative distribution functions of normalized read counts for each gRNA in ACE2-sorted populations in 3 independent biologic replicates of the focused secondary CRISPR screen of wild-type (A) and ACE2-enriched (B) HuH7 cells. In each subpopulation, >99.9% of all possible gRNAs were detected and the ratio between the 90th and 10th percentile most abundant gRNAs was <10. (C-D) Mean log2 fold-change of each individual gRNA targeting ACE2, the aggregate mean of these individual ACE2-targeting gRNAs, and a violin plot of the distribution for all 75 control nontargeting gRNAS (lines indicate quartiles) in the focused ACE2 CRISPR screens of wild-type (C) and ACE2-enriched (D) HuH7 cells. (E-H) Q-Q plots of observed versus expected–log(p) for positive (E,G) or negative (F, H) regulation of ACE2 surface abundance for every gene in the focused CRISPR screen of HuH7 wild-type (E-F) or ACE2-enriched (G-H) cells. Observed p-values were calculated by MAGeCK gene-level analysis. (TIF) [file ppat.1010377.s003.tif]

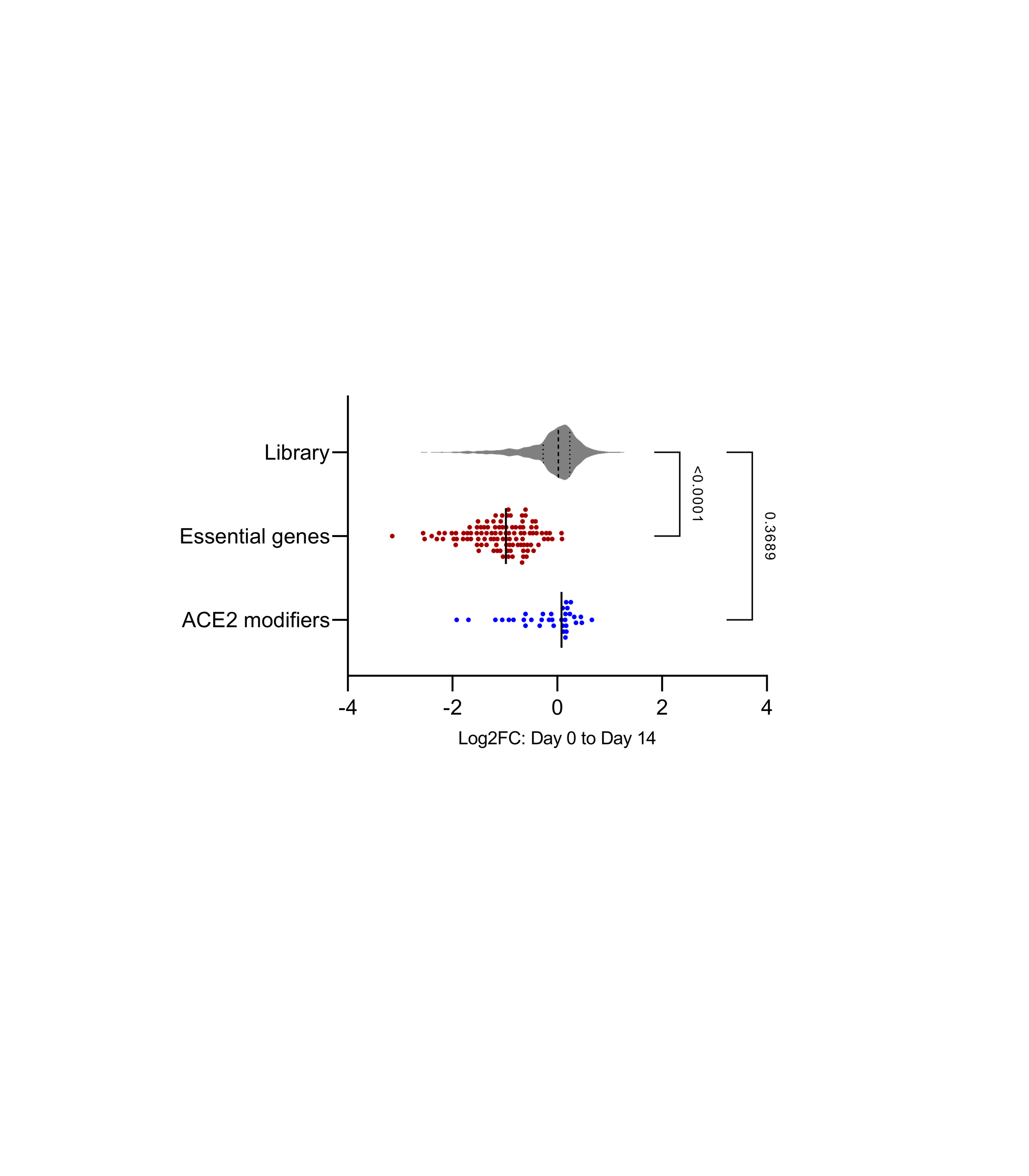

Supplement: S4 Fig — Individual gRNAs were quantified in the starting CRISPR library plasmid pool and the HuH7 wild-type screen day 14 FACS input. A violin plot is displayed for all genes in the library, along with individual data points for genes identified as Tier 1 Core Essential Genes in human cell lines [60] or identified as HuH7 ACE2 modifiers in this study. Lines indicate median values. Annotated p-values were calculated by Fisher’s LSD test. (TIF) [file ppat.1010377.s004.tif]

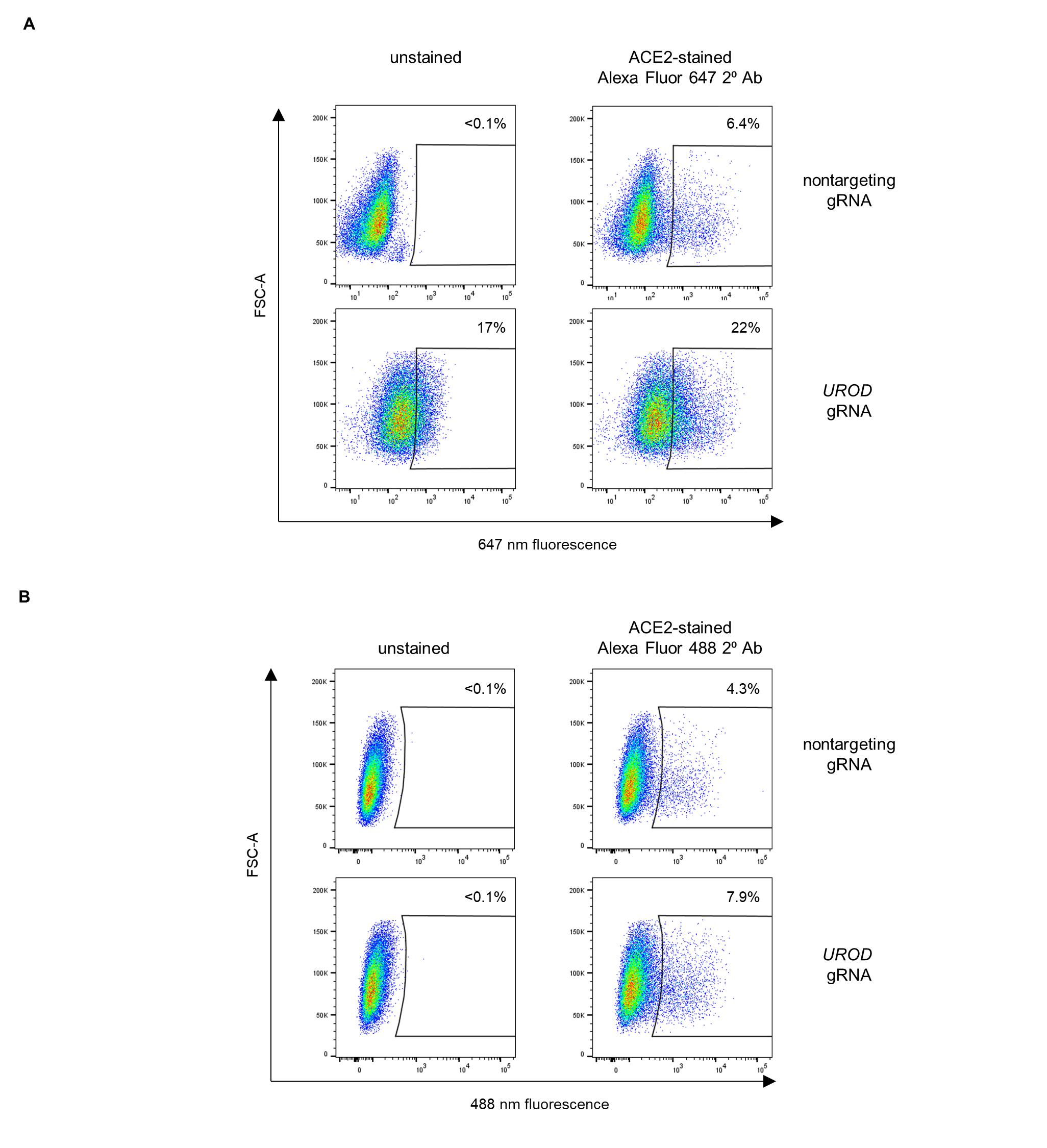

Supplement: S5 Fig — (A-B) FACS plots of fluorescence intensities of control and UROD-targeted HuH7 cells either unstained or incubated with an ACE2 antibody and a corresponding Alexa Fluor 488 (A) or Alexa Fluor 647 (B) conjugated secondary antibody. (TIF) [file ppat.1010377.s005.tif]

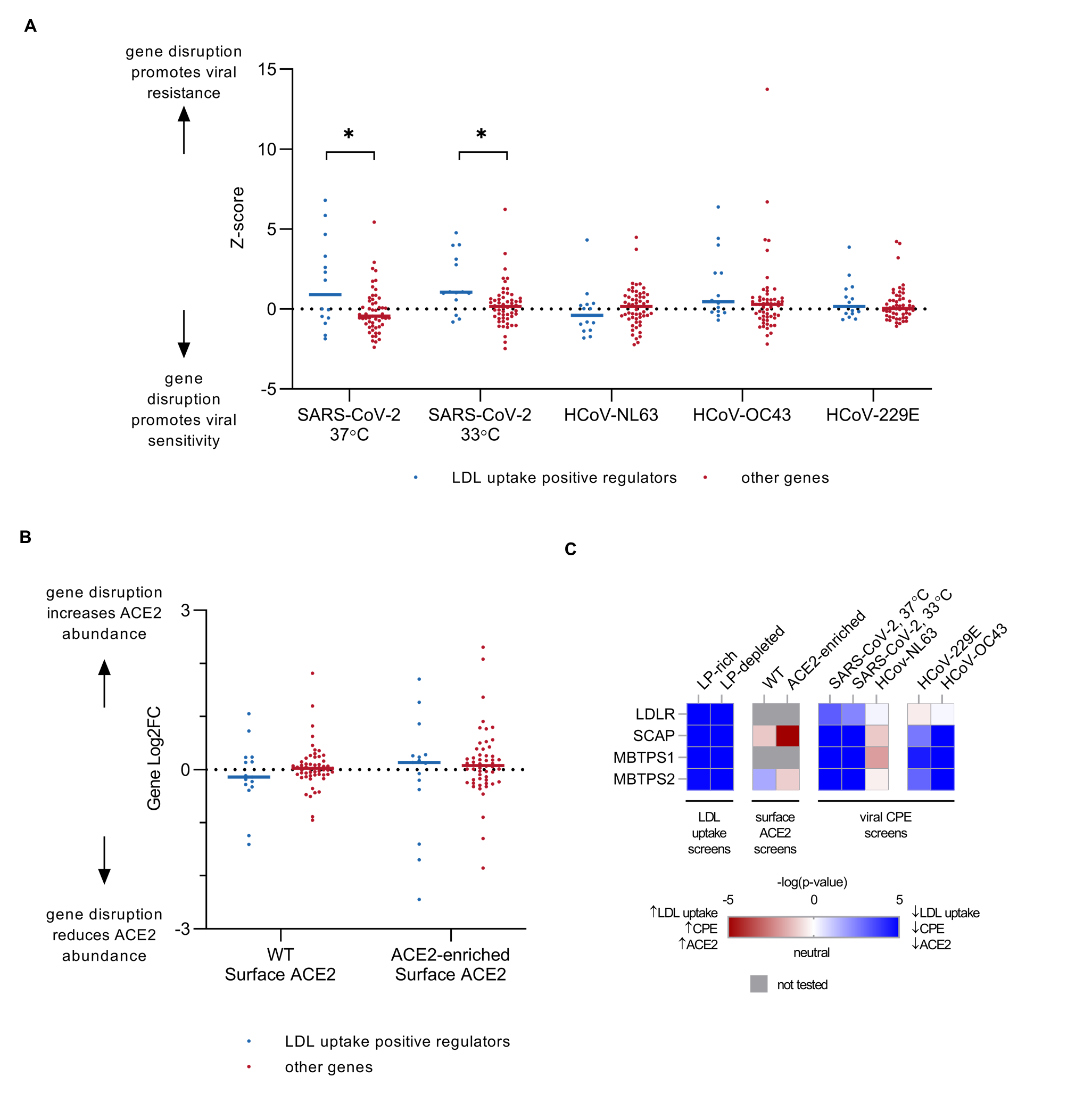

Supplement: S6 Fig — (A) The subset of genes tested by high-resolution CRISPR screening for both LDL endocytosis and ACE2 surface abundance were divided into those whose disruption reduced LDL uptake (n = 15) and “other genes” whose disruption did not reduce LDL uptake (n = 55). The Z-score for each gene within each group was then compared for each of the viral CPE screens reported by Schneider et al. Asterisks indicate p < 0.05 by Student’s t-test. (B) Genes were grouped as in (A) and compared for their association with surface ACE2 abundance in secondary screens of either HuH7 wild-type or ACE2-enriched cells. (C) Heat maps for log-transformed p-values of canonical SREBP regulators LDLR, SCAP, MBTPS1, and MBTPS2 in screens of HuH7 LDL uptake [43] and ACE2 surface abundance (this study) and HuH7.5 SARS-CoV-2 cytopathic effect [35]. (TIF) [file ppat.1010377.s006.tif]

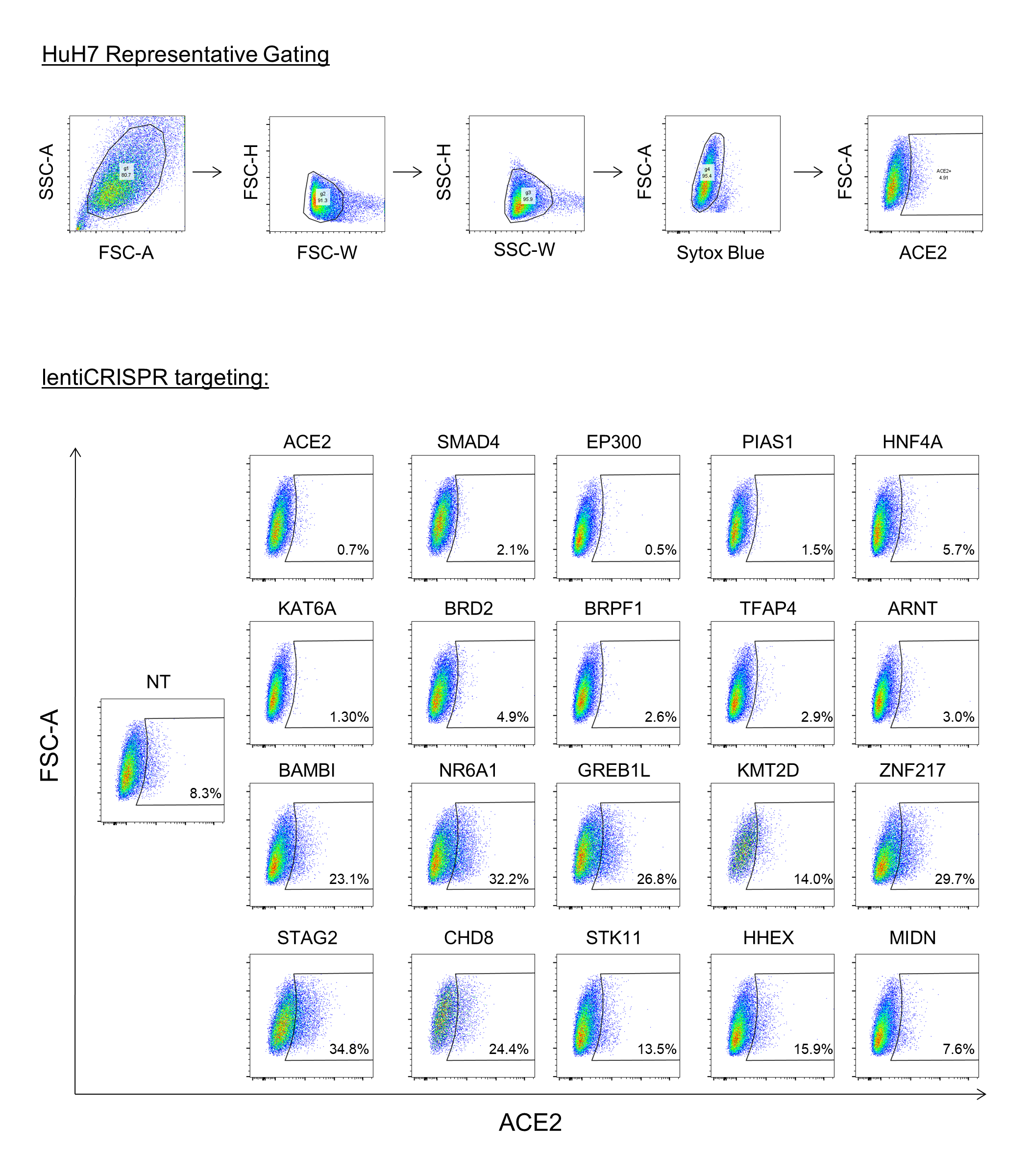

Supplement: S7 Fig — FACS plots of gating strategy and ACE2 staining relative to forward scatter area for untreated and single gRNA CRISPR-targeted wild-type HuH7 cells. FACS plots for a representative replicate of Fig 2A are displayed. Raw data for all replicates and statistical analysis are provided in S9 Table. (TIF) [file ppat.1010377.s007.tif]

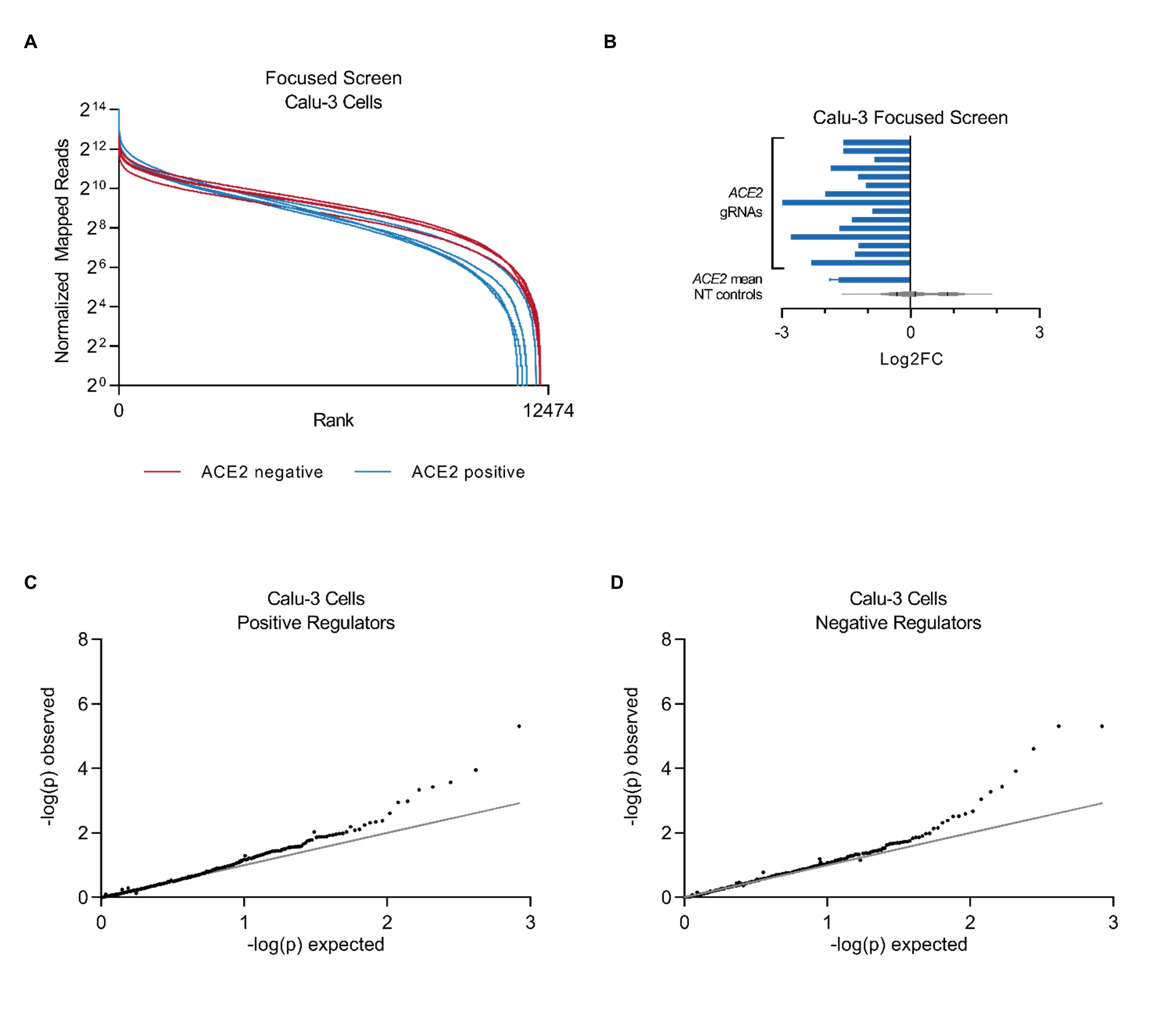

Supplement: S8 Fig — (A) Cumulative distribution functions of normalized read counts for each gRNA in ACE2-sorted populations in 3 independent biologic replicates of the focused CRISPR screen of Calu-3 cells. (B) Mean log2 fold-change of each individual gRNA targeting ACE2, the aggregate mean of these individual ACE2-targeting gRNAs, and a violin plot of the distribution for all 75 control nontargeting gRNAS (lines indicate quartiles). (C-D) Q-Q plots of observed versus expected–log(p) for positive (C) or negative (D) regulation of ACE2 surface abundance for every gene in the focused CRISPR screen of Calu-3 cells. Observed p-values were calculated by MAGeCK gene-level analysis. (TIF) [file ppat.1010377.s008.tif]

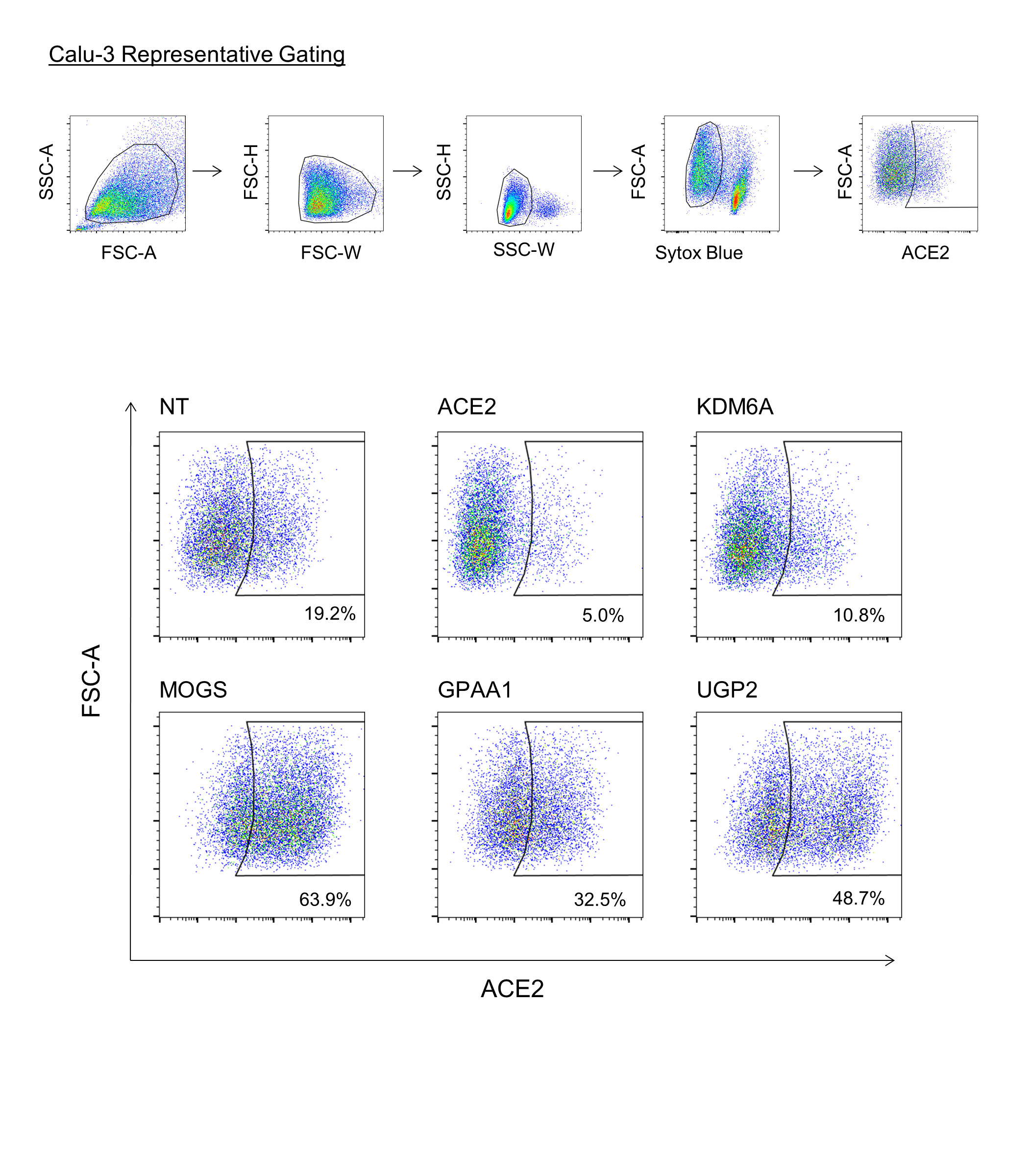

Supplement: S9 Fig — FACS plots of gating strategy and ACE2 staining relative to forward scatter area for untreated and single gRNA CRISPR-targeted Calu-3 cells. FACS plots for a representative replicate of Fig 6E are displayed. Raw data for all replicates and statistical analysis are provided in S9 Table. (TIF) [file ppat.1010377.s009.tif]
