# Supplementary material for: Identification of cell type specific ACE2 modifiers by CRISPR screening
Source: PLoS Pathog. 2022 Mar 1;18(3):e1010377. doi: 10.1371/journal.ppat.1010377 (PMC8929698; doi:10.1371/journal.ppat.1010377)
Supplement: Attachment — Submitted filename: point-by-point.docx [file ppat.1010377.s020.docx]

**Title: Identification of cell type specific ACE2 modifiers by CRISPR screening**

We thank the editor and both reviewers for their thoughtful evaluation of our initial submission. The decision letter and reviewer comments concisely and accurately described the key findings and limitations of our manuscript. This review identified several opportunities for improvement, which we have addressed with additional experimentation, analysis, and discussion. These revisions are detailed in a point-by-point response below. In summary:

1. We experimentally tested the functional effect of HuH7 ACE2 modifiers in a second cell line, Calu-3 respiratory epithelial cells. These cells have been previously found to serve as good models for the viral replication kinetics and interferon response of primary airway epithelial cells (Rebendenne et al, *J. Virol.* 2021, PMID 33514628). Surprisingly, we found that *SMAD4* and other HuH7 ACE2 modifiers did not have a similar effect in Calu-3 cells.
2. Given this lack of generalizability between cell lines, we performed an additional CRISPR screen for modifiers of ACE2 surface abundance in Calu-3 cells. Although *ACE2* was identified as the top hit in Calu-3 cells (as it was in HuH7 cells), there was otherwise very limited overlap between the genes identified, again consistent with a high degree of cell type specificity in ACE2 regulatory networks.
3. We performed single gene validation studies of Calu-3-specific ACE2 modifiers, confirming a functional effect for 5/5 genes tested (*ACE2*, *KDM6A*, *MOGS*, *GPAA1*, *UGP2*).
4. In several sections, we have provided additional experimental controls, methodological details, and expanded discussion of our findings. These changes are outlined below.

We are grateful for this process, which we believe has resulted in a greatly improved manuscript.

**Reviewer #1:**

**In this manuscript, Sherman and co-workers present a CRISPR platform to screen for human genes that modulate ACE2 expression. ACE2 is the cellular receptor for a few human coronaviruses, i.e. SARS-CoV-1, SARS-CoV2, and HCoV-NL63. The authors hypothesized that some of the previously reported SARS-CoV-2 host factors (validated or not) might be involved in regulating ACE2 (SARS-CoV-2 receptor) expression. They have selected ~800 of these host factors as targets for engineering a custom-designed CRISPR KO guide RNA library. To screen for host genes that could potentially regulate ACE2 expression, the authors choose a human hepatoma cancer cell line, Huh7, that is known to be a susceptible & permissive cell line to SARS-CoV-2. Two different populations of Huh7 cells were used – an original population that consists of only a small % of cells expresses ACE2 and an ACE2-enriched population (pre-sorted by flow cytometry). These two different populations of Huh7 cells were mutagenized by the custom CRISPR library, allowed to proliferate, and later sorted by flow cytometry to harvest ACE2 positive/negative cells. Using NGS and bioinformatics, the authors identify 35 candidate genes from the screen and subjected 20/35 of these genes for validation (i.e. measuring % of ACE2 positive cells present in each genetically perturbed cell population). Most of these genes (but not all) were validated as ACE2 levels seemed to be altered upon CRISPR KO in Huh7 cells (both original population and ACE-enriched population). Using pathway analyses – enrichment of GO terms (Molecular Functions) and STRING, the authors suggested that some of the validated hits and some yet-to-be validated hits from the screen may transcriptionally regulate ACE2 expression in the Huh7 cells. Compared to published datasets, the authors propose establishing a correlation between genetic perturbations of a few validated and yet-to-be validated hits on this screen, and resistant phenotypes against SARS-CoV-2 and HCoV-NL63. The authors further examine the ACE2 protein levels in a few CRISPR KO Huh7 cell populations (not sure if this was the original or ACE2-enriched population) by immunoblotting and immunofluorescent imaging, as well as determine the % of ACE2 positive cells by flow cytometry. ACE2 protein/mRNA levels seemed to be dramatically reduced upon depletion of SMAD4, EP300, and PIAS1. In contrast, more abundant ACE2 protein/mRNA was observed in BAMBI KO cells. Subsequently, they demonstrate SARS-CoV-2 infection and propagation in all of these KOs (again, not sure if these derived from the original Huh7 or ACE2-enriched Huh7), except BAMBI, were significantly reduced. BAMBI KO, in contrast, promoted SARS-CoV-2 infection and propagation.

Overall the manuscript is well written, but some hiccups are spotted. For example, a few supplementary figures are either provided without being mentioned in the text (e.g. Fig S2, 3E-3H & S4) or inaccurately cited in the text (e.g. Page 8: “We observed that gene disruptions which reduced LDL uptake were also more likely to confer cellular resistance to SARS-CoV-2 both at 37⁰C and at 33⁰C (Figure S3A).” Please check the first paragraph of Page 9, too).**

We thank Reviewer #1 for this favorable summary of our findings, and for highlighting several points requiring further clarification. We have now provided additional detail in the Figure Legends to clarify when we analyzed HuH7 wild-type or serially ACE2-enriched cells for each analysis. We have also carefully reviewed all references to supplemental and main figures and tables to ensure accuracy.

**Major comments:**

1. **The authors cited Wei Jin et al. (2021, Cell) in the manuscript without mentioning a key finding in the article – HMGB1 as a novel regulator of ACE2 expression and a critical factor for SARS-CoV-1, SARS-CoV-2, and HCoV-NL63 infections. Please explain the reason(s) for omitting this important discovery, which is perfectly relevant to this manuscript.?**

Although HMGB1 depletion was shown by Wei et al. to affect ACE2 expression in Vero E6 cells, a similar experiment was not performed in either HuH7.5 or Calu-3 cells. These findings therefore are not in direct contradiction to our study. However, Wei et al. did find HMGB1 depletion in HuH7.5 and Calu-3 cells to confer resistance to SARS-CoV-2 cytopathic effect (CPE), which indeed may be caused by decreased ACE2 expression. If this were the case, we suspect that the discrepancy between our studies arose from technical differences such as CRISPR library composition, cell culture conditions, or other experimental variables. Alternatively, HMGB1 depletion may have pleitropic effects, with the CPE phenotype observed by Wei et al. in HuH7.5 and Calu-3 cells mediated by an ACE2-independent mechanism. To address these points, we have added a specific discussion of *HMGB1* to the revised manuscript (page 15; please note all page numbers listed herein refer to the version of the revised manuscript without “track changes”).

2. **Validated genes (i.e. 18/20) – candidate genes considered as validated ACE2 regulators were not clearly defined in the text. Two sets of validations were presented (Fig. 2A and 2B), >2 genes from these validations were clearly shown to give negligible impact to influence % of ACE2 expressing cells upon knockout in original Huh7 and ACE2-enriched population. For instance, % of ACE2 positive for HNF4A KO, BRD2 KO, and ARNT KO in the original Huh7 look the same as the WT/NT. For the ACE2-enriched population, BRD2 KO, TFAP4 KO, KMT2D KO, and ARNT KO did not seem to alter % of ACE2 expressing cells. Please clarify, and if possible, please provide statistical evaluations for data presented in Fig. 2A and 2B.**

To address these points, we have now added annotation of statistically significant results to Figure 2, methodologic details for the statistical analysis to the corresponding Figure Legend (pages 29-30), and raw data to Supplemental Table 9. In general, given the low basal expression of ACE2 in wild-type HuH7 cells, the effects of disrupting ACE2 modifiers were more clearly seen when cells were first serially enriched for ACE2 expression. We have also added data for mean fluorescence intensity in each cell line (Supplemental Table 9), which are broadly consistent with the analysis of ACE2-positivity.

3. **A significant number (9/20) of the top hits selected for validation in Fig. 2 has been previously identified as “strongly selective cancer dependency genes” (DepMap definition: “whose dependency is at least 100 times more likely to have been sampled from a skewed distribution than a normal distribution”) from a huge collection of CRISPR screens for cancer cell lines. For example,**

**1) PIAS1 (**[**https://depmap.org/portal/gene/PIAS1?tab=overview&characterization=expression**](https://depmap.org/portal/gene/PIAS1?tab=overview&characterization=expression)**)
2) HNF4A (**[**https://depmap.org/portal/gene/HNF4A?tab=overview&characterization=expression**](https://depmap.org/portal/gene/HNF4A?tab=overview&characterization=expression)**)
3) BAMBI (**[**https://depmap.org/portal/gene/BAMBI?tab=overview&characterization=expression**](https://depmap.org/portal/gene/BAMBI?tab=overview&characterization=expression)**)
4) KAT6A (**[**https://depmap.org/portal/gene/KAT6A?tab=overview&characterization=expression**](https://depmap.org/portal/gene/KAT6A?tab=overview&characterization=expression)**)
5) TFAP4 (**[**https://depmap.org/portal/gene/TFAP4?tab=overview&characterization=expression**](https://depmap.org/portal/gene/TFAP4?tab=overview&characterization=expression)**)
6) NR6A1 (**[**https://depmap.org/portal/gene/NR6A1?tab=overview&characterization=expression**](https://depmap.org/portal/gene/NR6A1?tab=overview&characterization=expression)**)
7) GREB1L (**[**https://depmap.org/portal/gene/GREB1L?tab=overview**](https://depmap.org/portal/gene/GREB1L?tab=overview)**)
8) ZNF217 (**[**https://depmap.org/portal/gene/ZNF217?tab=overview**](https://depmap.org/portal/gene/ZNF217?tab=overview)**)
9) HHEX (**[**https://depmap.org/portal/gene/HHEX?tab=overview&characterization=expression**](https://depmap.org/portal/gene/HHEX?tab=overview&characterization=expression)**)

As mentioned in the Materials and Methods, during the CRISPR screen for identifying ACE2 regulators, populations of mutagenized Huh7 cells (cancer cells) were cultured and allowed to proliferate for 14 days. Would it be possible that any of these “strongly selective” cancer dependency genes confound the interpretation on the CRISPR screen hits? Please discuss.**

Reviewer #1 raises an interesting point. While it is certainly possible that some of the identified ACE2 modifiers may affect cellular viability or proliferation, our screen selection does not depend on gRNA enrichment over time but rather the segregation of a gRNA between sorted populations at a single time point. Therefore, even if a particular gRNA slowed (or accelerated) the proliferation of edited cells, it should still be equally represented in ACE2^high^ and ACE2^negative^ populations at a single time point so long as it has no influence on ACE2 surface abundance.

To confirm this expectation, we have added a formal analysis of the influence of each ACE2 modifier on HuH7 cellular proliferation to our revised manuscript (Figure S4, Table S7). We sequenced our day 14 FACS input samples relative to our starting plasmid pool to systematically assess for a bias in ACE2 modifiers for genes affecting cell survival and proliferation. Our power to detect essential genes by this approach is demonstrated by our clear detection of gRNA depletion for genes identified as “Tier 1 Core Essential” genes in a recent analysis of the DepMap database (Pacini et al, *Nature Communications* 2021, PMID 33712601). For the hits from our ACE2 screen relative to the entire CRISPR library, we found no significant difference in gRNA enrichment or depletion over 14 days of passaging.

4. **STRING analysis – why was ACE2 excluded from the analysis? What criteria were set for the analysis, e.g. network type, meaning of network (evidence/confidence), active interaction sources (text-mining/experiments/databases/Gene Fusion/etc.)? What was the rationale for including invalidated hits (HNF4A, BRD2, TFAP4, and ARNT) in this analysis (and in Fig. 3A, 3C and 3D)?**

As discussed above, most screen hits (17/20) that were tested by single gene disruption were validated in at least 1 condition (parental wild-type or ACE2-enriched HuH7 cells). Among the additional 15 screen hits not tested individually, it is likely that some if not most of these have an impact on ACE2, and we therefore felt they should be included in the analysis of the screen results. We have added details for the STRING analysis to the revised Methods section (pages 18-19). For simplicity, we omitted nodes that were not connected to other nodes (like ACE2) in our visualization. We have clarified this point in the Figure 3 Legend.

5. **Promising results were demonstrated for mechanistic dissections of SMAD4, EP300, PIAS1, and BAMBI in regulating ACE2 expression, as well as SARS-CoV-2 infection. However, all of these efforts were performed based on the same cancer cell line, in fact, only a single cell line was used throughout the entire manuscript. In addition, these genes are depleted using a single guide RNA, without any cDNA rescue/addback to cross-validate and rule out any off-target effects. It would be crucial to strengthen these important findings by using additional cell lines/human primary cells, and additional guide RNA(s). All CRISPR KO Huh7 cells reported in this manuscript were generated using a lentivirus system, which involved random integration of Cas9 and guide RNAs into the Huh7 genome. Would that lead to an off-target impact? Would it be possible to transiently introduce Cas9 and guide RNAs into cells (without integration) to knockout these genes for cross-checking the impact on ACE2 expression?**

Both reviewers commented on the limitations of analysis restricted to a single cell line. To address this important point, we have now performed additional experiments examining ACE2 modifiers in Calu-3 respiratory epithelial cells (Figures 6, S8, S9; Tables S8, S9). We tested not only HuH7 ACE2 modifiers but all 833 genes target by the CRISPR library in another screen of Calu-3 cells. The rigor of this screen was supported by the identification of the expected control *ACE2* as the top hit and by the subsequent experimental validation of 4/4 additional Calu-3-specific ACE2 through single gene disruption. Unexpectedly, we identified distinct sets of ACE2 modifiers in either cell line. These results demonstrate the cell type specificity of ACE2 regulatory networks, which is now highlighted as an important finding from our study.

Regarding the potential confounding influence of nonspecific lentiviral integration, it is important to note that our CRISPR screens were performed at a scale of ~200-500 cells for each individual gRNA in the library. Therefore, although it is possible that lentiviral integration may nonspecifically disrupt a genomic site relevant to ACE2 regulation, it is highly unlikely to integrate into the same locus repeatedly in these ~200-500 cells. Any random integration event in a single cell will therefore comprise only a tiny fraction of the overall testing for a particular gRNA sequence. It is also highly unlikely that a nonspecific integration event would be similarly biased for multiple independent gRNAs targeting the same gene. For our screen hits, the enrichment or depletion in sorted populations is apparent for several independent gRNAs targeting the same gene (in most cases >10 of 15). The importance of on-target gRNA activity is further supported by the fact that we do not detect significant enrichment for control non-targeting gRNAs.

6. **It is reassuring to see SARS-CoV-2 infection, production, and spread was blocked/promoted in the KO cells at 48 hpi, after multiple rounds of infection. However, the role of these genes in regulating ACE2-mediated entry of the virus was not specifically tested. This key point could perhaps be addressed by standard virus entry assays using either a surrogate system (BSL2) such as recombinant VSV expressing Spike protein of SARS-CoV-2, or HCoV-NL63 (ACE2 dependent, BSL2 pathogen). No additional BSL3 work is required.**

We agree with Reviewer #1 that analysis of virus entry would be of interest. However, our initial attempts to test pseudotyped viral entry in control and CRISPR-edited HuH7 cells were limited by a very weak luciferase signal from our reporter virus. We were able to achieve higher levels of signal through heterologous overexpression of ACE2 in target cells, but this system utilizes an ectopic promoter and is not expected to be sensitive to regulators of the endogenous *ACE2* locus. Although we believe it is unlikely that HuH7 ACE2 modifiers are affecting SARS-CoV-2 infection in a manner distinct from ACE2-mediated entry, this possibility cannot be excluded.

**Minor comments:**

7. **Please label the protein standard for all western blots.**

We have added protein standards to all western blots (Figures 1C, 4A, 6E).

8. **Page 6: “In accordance with the depth of library coverage in this screen, we found >99.9% library representation in each sorted cell population with minimal skewing of gRNA representation.” Is “>99.9%” referred to the entire library against “833 selected genes”? This statement is pretty confusing as each sorted cell population should theoretically consist of a distinctive gene pool since each group exhibited different levels of ACE2 expression (i.e. ACE2 negative or positive). Perhaps I simply misunderstood the overall design of the screen and/or the statement.**

Although the screen does rely on the enrichment or depletion of individual gRNAs into either sorted subpopulation, this is not an “all-or-none” phenomenon. This is reflected by the internal control *ACE2*, for which targeting gRNAs are not absent in the ACE2-positive subpopulation but rather depleted. The incomplete segregation of gene-targeting gRNAs may occur for a variety of reasons. Not all of the ~200-500 cells containing a specific gRNA will undergo editing with a loss-of-function indel at all alleles for a given gene. Therefore, cells with unedited or hypomorphic alleles may not segregate completely between the sorted populations. Also, even if every cell with a specific gRNA is edited with complete loss-of-function alleles, the resulting effect on ACE2 may be to simply shift its Gaussian distribution, such that the upper or lower tail of that distribution still falls within either FACS gate.

Regarding the QC analysis for the library synthesis, the “>99.9% library representation” refers to the proportion of individual gRNAs in the entire library that were detected in each subpopulation. The “minimal skewing” refers to the ratio of counts for the 90^th^ and 10^th^ percentile most abundant gRNAs. These measures are part of our routine analysis of CRISPR screen sequencing data, as underpowered screens with inadequate depth of coverage can be detected through lower proportions of gRNA representation and/or increased skewing (higher ratios of 90^th^/10^th^ percentile counts) after an experimental bottleneck (as was the case in our primary genome-wide screens). We have revised the Results (page 6), Methods (page 18), and corresponding Figure S2, S3, and S8 Legends (pages 32-33, 35) to more clearly describe these analyses.

9. **Pg. 18 “Immunobloting of Huh7 RIPA lysates……. against….. SMAD4, EP300, PIAS1” data not shown in the results. Please either share the results or revise the Materials & Methods. Totally understand that the authors may have gone through multiple rounds of manuscript prep; please check through again as multiple hiccups have been spotted.**

We thank Reviewer #1 for identifying this error. We initially had difficulty finding adequate commercial antibodies for immunoblotting of these proteins to confirm target depletion. In this revision, we have now confirmed protein depletion for 4/5 HuH7 targets and 5/5 Calu-3 targets, with immunoblots included in the revised manuscript (Figures 4A and 6D).

**Reviewer #2:**

**In their manuscript, “Identification of ACE2 modifiers by CRISPR screening,” Sherman et al. aim to identify genes that modify ACE2 surface expression in human hepatocarcinoma cells. SARS-CoV-2, the etiological agent of COVID-19, requires surface expression of ACE2 for cell entry and for productive infection, making modulation of ACE2 expression an enticing therapeutic target. In the hepatoma cells investigated here, as in other human tissues, ACE2 expression levels are heterogenous, and the genetic/epigenetic regulators of these expression patterns are incompletely defined. By employing a set of CRISPR Loss-of-Function screens, the authors identify 35 candidate genes that potentially modulate ACE2 surface expression. In subsequent validation experiments, the authors validate the effects of a subset of screen hits and implicate central mediators of the SMAD4 signaling axis in regulating ACE2 expression. Finally, CRISPR-mediated gene deletion of several top hits is employed to demonstrate functional consequences on the ability of SARS-CoV-2 to productively infect HUH-7 cells.
The studies detailed in the manuscript address a significant research question, with implications for potential therapeutic strategies. The rationale for focused CRISPR screens is clearly explained, and analyses are appropriately implemented. Validation experiments are properly controlled and confidently ascribe ACE2 expression regulatory effects to multiple screen hits. Previously published data is used extensively to correlate ACE2 screen hits with the effects of gene deletion on SARS-CoV-2 infection (Schneider et al., 2021), and LDL uptake (Emmer et al., 2021). However, despite convincing identification and validation of cellular factors that modulate ACE2 expression, there are significant concerns regarding the generalizability of the findings. Moreover, while comparisons to published screens provide important context for the current work, attempts to directly correlate per gene effects may not be fully appropriate due to differences in cell lines and experimental systems. While the SMAD4 signaling axis appears to play a significant role in modulating ACE2 expression, elucidating underlying mechanisms would require additional experimentation. Finally, the manuscript is missing numerous important methodological details and figure annotations. Specific major and minor points are detailed below.**

We thank Reviewer #2 for this insightful summary of our findings and opportunities for improvement. We have responded to the comments on generalizability, mechanism, methodology, and figure annotation in our responses to the specific points below.

**Major points:**

1. **Cell line used and generalizability of results: The Huh7 hepatoma cell line seems to recapitulate the sporadic nature of ACE2 expression in human tissues, making it a good choice for the CRISPR screens described here. In addition, the “two pronged” screen approach (using “non-enriched” and ACE2 expression-enriched populations) effectively improves robustness and hit detection. However, despite comparisons to published data, there is limited evidence provided that the described hits are likely to modulate ACE2 surface expression in additional (and perhaps more physiologically relevant for SARS-CoV-2 infection) cell lines. Additional gene deletion experiments for top screen hits in other cell types are needed to evaluate the generalizability of these effects. Potentially informative cell types include line with high ACE2 expression (e.g., HepG2 cells) and low ACE2 expression (e.g., A549), or perhaps primary airway epithelial cells (as a physiological target of SARS-CoV-2 infection – although this may be beyond the scope of the present manuscript). In addition, interpretation of screen results would benefit from additional characterization of the Huh7 cell lines used. Specifically, the stability of ACE2 expression in serially-enriched ACE2 cells should be assessed. Given that the guide enrichment/depletion is evaluated in populations FACSorted based on ACE2 expression, “baseline” instability of ACE2 expression, particularly in the enriched group, might impact screen results.**

Both reviewers highlighted the important limitation of analyzing only a single cell line in our original manuscript. As described in response to point 5 by Reviewer #1, we have now performed additional experimentation to interrogate the functional modifiers of ACE2 abundance in Calu-3 respiratory epithelial cells (Figures 6, S8, S9; Tables S8, S9).

Regarding the dynamics of ACE2 expression over time in HuH7 cells serially enriched for ACE2, this characterization was included in a recent manuscript from our lab (Sherman and Emmer, *Scientific Reports* 2021, PMID 34354120). In brief, ACE2 expression is quasi-stable in these cells, with daughter cells of ACE2-expressing cells maintaining higher levels of ACE2 expression but gradually reverting back to the starting proportion of 3-5% ACE2-positivity with continued passaging in culture. Like Reviewer #2, we had suspected that ACE2 enrichment prior to screening might impact the screen results, perhaps by improving detection for positive regulators of expression, with parental cells (with lower baseline ACE2 expression) better suited to identify negative regulators. Surprisingly, we found very similar results with either approach (Figure 1F).

2. **Proposed mechanisms of ACE2 regulation: Screen results and validation experiments implicate SMAD4 signaling components in regulation of ACE2 surface expression. Although the manuscript includes studies demonstrating that these effects operate at the level of mRNA transcription, the underlying mechanistic details are not explored. With the convincing results for SMAD4, additional investigation of TGFbeta/BMP signaling pathways could be investigated for effects on ACE2 expression levels, with different pathway agonists that activate different SMAD complexes evaluated for effects on ACE2 transcription. Whether or not the effects of SMAD4 are direct or indirect could be explored with analyses of the ACE2 promoter and/or SMAD4 binding. Given the diverse roles of SMAD4 (and other focused hits) on cell biology, it would also be of interest to assess how SMAD4 disruption (and disruption of additional related screen hits) affects transcription more generally (e.g. through RNA-Seq comparisons), that might also point towards indirect effects on ACE2 expression and/or SARS-CoV-2 infection efficiency.**

We thank Reviewer #2 for these insightful comments and agree that the suggested experiments would clarify the mechanism of SMAD4 regulation of ACE2. Our original intent was to indeed pursue a deeper investigation of SMAD4 functional regulation of ACE2 expression with candidate ligand and receptor testing, SMAD4 ChIP-seq, and transcriptome analysis of SMAD4-depleted cells. However, given our interim findings on the cell type specificity of ACE2 regulatory networks, we have since prioritized the mechanistic investigation of ACE2 modifiers in respiratory epithelial cells, which are likely to be more relevant to lung pathology in COVID-19. These studies remain in their early stages, however. We do note that a recent study (Chen et al., *Cell Mol Gastroenterol Hepatol* 2021, PMID 32810597) reported SMAD4 ChIP-seq from intestinal epithelial cells, indeed finding evidence of SMAD4 binding at the *ACE2* promoter. A discussion of these findings is included in our revised manuscript (pages 14-15).

3. **Confirmation of CRISPR-mediated gene disruption: Experimental design and well controlled experiments strongly suggest that disruption of multiple major screen hits alters ACE2 surface expression levels. However, with the exception of ACE2, there are no data presented that validate the efficiency of CRISPR-mediated gene deletion. The efficiency of gene depletion should be confirmed for focused hits, and ideally presented alongside with corresponding ACE2 expression**.

As noted in response to point 9 by Reviewer #1, we have added immunoblots confirming efficient protein depletion induced by CRISPR editing for 9/10 target genes (Figures 4A, 6D).

**Minor points:**

4. **While a schematic of screen design and interpretation is helpful in understanding the work, as presently constructed, Figure 1A does not clearly portray the screening methodology.**

We have revised the schematic to portray the screen design (Figure 1A) more clearly and added methodologic detail to the corresponding Figure 1 Legend (page 29) and Methods (pages 17-18).

5. **Figure S2 C-D, Figure S3 C-D: QC results should include log2 fold-change values for negative controls in the guide library.**

We have revised these figures (Figures S2, S3, S8) and their corresponding Figure Legends (pages 32-33, 35) to include data on nontargeting control gRNAs for all screens.

6. **Both whole genome and focused CRISPR screens were properly conducted in multiple biological replicates. However, how these replicates were pooled and analyzed is not described. Relatedly, while the Methods section contains details on the initial whole genome screen, it appears that a description of the focused screen has been omitted.**

We have revised the Methods section to correct this oversight (pages 17-18). We have also added annotation to the corresponding raw data (Tables S3-S6, S8) to clarify the statistical analysis of the CRISPR screens.

7. **CRSIPR screen supplementary tables should include a legends and explanations for the different columns**

As discussed above, we have revised these supplementary tables to include this information (Tables S3-S6, S8).

8. **Validation experiments, Figure 2, S6, S7: Results as presented indicate the degree to which gene deletion impacts the proportion of ACE2-posiitve cells. It would be informative to also assess and present how deletion affects the abundance of ACE2 on cells (e.g. by mean fluorescence intensity).**

We have added data for ACE2 mean fluorescence intensity (MFI) to Supplemental Table 9. Because these replicates were performed on separate FACS runs with different instrument settings, we cannot directly compare absolute MFI across replicates. We have therefore normalized these values to nontargeting controls for each replicate. We also note that in HuH7 wild-type cells, only a small proportion of cells express detectable ACE2, and so an average of MFI across the entire cell population (ACE2 expressing and non-expressing cells) has very limited signal above background. This is reflected in the nonsignificant value for the control *ACE2*-targeting gRNA, despite these same cells exhibiting a clear reduction in the proportion of ACE2-expressing cells (Figures 2A and S7, Table S9). In ACE2-enriched cells, by contrast, baseline ACE2 expression is much higher, and MFI changes upon single gene disruption more closely correlate with the analysis of ACE2-positivity (Figure 2B, Table S9).

9. **Figure 2 does not report statistical tests. As a result, it is unclear which genetic knockouts result in statistically significant changes to ACE2 expression. In addition, in ACE2-enriched cells, data points for replicate 1 and replicate 3 appear to be very similar, while this is not the case for replicate 1 and replicate 3 measurements in non-enriched Huh7 cells. It should be made clear if these are technical or biological replicates.**

We have added details for all statistical tests to the corresponding Figure Legends. The astute observation by Reviewer #2 on the similarities between replicates 1 and 3 led us to reexamine our FACS analysis. We had inadvertently drawn the ACE2-positive gate more restrictive on replicate 2. We have repeated this analysis (Figures 2, S7) and included all raw data (Table S9) in the revised manuscript. Although the major conclusions from this experiment have not changed, the revised analysis is a more accurate reflection of the data. We have also added data for mean fluorescence intensity of the entire population (Table S9), which is not dependent on the ACE2-positive gates, and broadly supports the conclusions of this analysis.

10. **Representative flow cytometry gating schemes should be included as supplemental figures. Moreover, ACE2 flow cytometry gates were set based on unlabeled cells; Given the many problems associated with ACE2 antibody surface labeling previously reported by the authors, was there an isotype control included for setting gates and/or assessing non-specific labeling?**

We have added representative flow cytometry gating schemes to the FACS plots (Figures S7, S9). In initial pilot experiments we had performed comparisons to isotype controls, finding similar distributions for unlabeled cells, cells stained with secondary antibody only, or cells stained with isotype control primary antibody with the corresponding secondary antibody. This analysis is included in our recent publication describing optimization of our ACE2 FACS protocol (Sherman and Emmer, *Scientific Reports* 2021, PMID 34354120). For simplicity, then, in our screen we used unstained cells to define ACE2-positive gates. For single gene disruption studies, we always included *ACE2*-targeted cells as controls for specificity of staining. The marked reduction in ACE2-positive cells upon *ACE2* targeting strongly supports that this staining is reflective of ACE2 surface expression rather than nonspecific primary or secondary antibody binding.

11. **For experiments presented in Figure 2, it is not clear if the cells were permeabilized prior to staining. The preprint cited in the text does include the methodological details.**

These cells were not permeabilized prior to staining and therefore reflect surface ACE2 abundance, as was also performed for the CRISPR screening protocol. This detail has been added to the corresponding Figure 2 Legend (pages 29-30) and Methods (page 20).

12. **The comparison of screen results to Schneider et al screen data presented in Figure 3C is missing several important annotations and methodological details that make interpretation challenging. It is unclear what values are plotted on heatmaps in Figure 3C – the color scale should be marked accordingly. Moreover, the exact source of values from Schneider et al. and how the comparisons were conducted should be reported.**

Heatmaps derived from the Schneider et al. study were generated using log-transformed p-values from the CRISPR screen on SARS-CoV-2 cytopathic effect in HuH7.5 cells. We have added annotation to Figure 3C and clarified this detail in the corresponding Figure Legend (page 31) and Methods (page 20).

13. **References to figure S4 are missing from the text.**

We had mistakenly referenced this figure as Figure S3 in the original manuscript. We have corrected this error in the revised manuscript.

14. **Figure S4A: what genes are considered to be “other genes”?**

“Other genes” refers to those tested in our prior LDL uptake CRISPR screen (Emmer et al, *PLOS Genetics* 2021, PMID 33513160) that did not affect LDL uptake by HuH7 cells. We have clarified this detail in the corresponding Figure Legend (page 34) and Results (page 9).

15. **Figure 4B should include statistical testing. Furthermore, additional clarification on the complementary methods used for figures 4B-D should be provided in the corresponding text.**

We have added statistical testing to Figure 4B and experimental details for Figures 4B-D to the corresponding Figure Legend (pages 30-31) and Methods (pages 19-20).

16. **Micrographs in Figure 4C should include a color key and scale bars. Furthermore, as only 3-5% of these cells (controls) are expected to express ACE2 (as reported), an analysis of multiple fields and methodological details on how they were selected should be included.**

These images were derived from the HuH7 cell line that had first undergone serial enrichment for ACE2 expression. Image acquisition and analysis of randomly selected fields was performed by a blinded observer. These details, along with a color key and scale bars, have been added to Figure 4C, its corresponding Figure Legend (page 32), and Methods (pages 20-21).

17. **In Figure 4D, it is unclear why the y-axis contains a “break” between identical values – perhaps a log10 scale without a break would be equivalent? In addition, the corresponding Methods section should indicate how these values were calculated, and additional details on the panel of control genes used normalization.**

We had included this break in the y-axis in an attempt to more clearly visualize the reduction in normalized mRNA levels <1. We have now reformatted this in Figure 4D. We have also added details regarding the quantification of qPCR values in the corresponding Figure Legend (pages 30-31) and Methods (page 21).

18. **Figure 5A describes the functional outcome of ACE2 reduction after CRISPR gene disruption on SARS-CoV-2 infection. While an M.O.I. of 48 was used for infection, the percentage of infected cells at 48 hours is unexpectedly low. This may be due to virus titration on a more permissive cell line (i.e. Vero-E6).**

We agree that the proportion of SARS-CoV-2-infected HuH7 cells observed may reflect the MOI calculations having been performed in Vero E6 cells. Additionally, heterogeneity of ACE2 staining may result in only the subset of ACE2-positive cells being infected. The importance of ACE2 expression to this readout is further supported by our finding of an increased percentage of infected cells upon BAMBI depletion (which increases the proportion of ACE2-expressing cells).

19. **The data presented in Figure 5A were collected by high-throughput microscopy on 384 well plates. Specific methodological details, including how images were obtained, how many fields were examined, magnification settings, and the analysis workflow used to calculate infection percentages should be provided.**

We have added these details to the revised Methods (page 21).

20. **In general, the Methods section should be significantly expanded, with additional details for each experimental approach and analysis presented.**

We have provided additional details in the revised Methods section as outlined in the responses to the specific points above.
