# Supplementary material for: Identification of cell type specific ACE2 modifiers by CRISPR screening
Source: PLoS Pathog. 2022 Mar 1;18(3):e1010377. doi: 10.1371/journal.ppat.1010377 (PMC8929698; doi:10.1371/journal.ppat.1010377)
Supplement: Attachment — Submitted filename: point-by-point.docx [file ppat.1010377.s021.docx]

**Title: Identification of cell type specific ACE2 modifiers by CRISPR screening**

We thank the editor and both reviewers for their continued thoughtful evaluation of our manuscript. The changes in our resubmission are detailed in a point-by-point response below.

**Reviewer #1:**

**Sherman and co-workers have addressed almost all reviewers’ comments and revised the manuscript. The key message for the revised version is that these ACE2 modifiers are likely to be cell type specific, as demonstrated by a new screen using an additional cell line (Calu-3). The authors have clearly stated the limitation of the findings in the revised manuscript.

Minor comments:

Fig. 2A and 2B
Good to see that statistical evaluations have been included. However, the data points plotted for each gene look different from the original submission. Also, data points for WT are removed. Is the original dataset used for these panels replaced by a newer version? Please clarify.**

Due to the astute review of our original submission, we had noticed that our original analysis had inadvertently applied gates for ACE2 positivity that were more restrictive for replicate 2 than for replicates 1 and 3. For our resubmission, we therefore repeated the analysis of the original flow cytometry data with careful attention to consistency of gating across all 3 replicates. This re-analysis was presented in our resubmission (Figures 2) along with the corresponding raw data (Figure S7, Table S9). We also added data for mean fluorescence intensity of the entire cell population for each sample (Table S9), which is not dependent on the setting of the ACE2-positive gates. Although the major conclusions from this experiment have not changed, the revised analysis is a more accurate reflection of the data.

**Fig 3
As shown in Fig 2, BRD2, MIDN, and ARNT (the 3/20 invalidated hits) do not impact ACE2 expression in any cell lines. It is puzzling to see these genes appearing in Fig 3 as “validated modifiers” of ACE2. Would it make more sense to exclude these invalidated hits in Fig 3? If not, please justify.**

The analysis in Figure 3 was completed prior to our single gene validation studies and included every gene identified by the CRISPR screen, of which a small fraction did not pass validation testing. These results do not completely rule out an effect of these genes on ACE2 expression. For example, *BRD2* targeting caused a 30-40% decrease in ACE2 positivity that did not meet the threshold for statistical significance; it is possible that additional replicates and increased statistical power may reveal a statistically significant effect. Even if this were the case, however, the effect size is likely to be small and we agree that these genes should be removed from Figure 3. *BRD2*, *MIDN*, and *ARNT* have now been removed from Figure 3 in this resubmission.

**Fig 5
A standard practice to rule out off-target effects is to complement KO cells with respective cDNAs and see if ACE2 expression/virus infectivity can be restored. Perhaps it would be challenging to fine-tune the expression of these ACE2 modifiers ectopically. Have the authors at least looked into that? If not, please justify and state the limitation (i.e., potential off-target effects).**

We thank reviewer #1 for this suggestion. We agree that complementation experiments could confirm that the phenotype caused by a given gRNA is due to on-target activity. The scale of our validation experiments, with 25 different genes individually tested, makes systematic cDNA expression for each challenging. It is important to note that our screen included functional testing of 15 unique gRNAs per gene, and that the genes identified as ACE2 modifiers demonstrated significant enrichment for the majority of these 15 independent gRNAs. While each of these gRNAs likely has significant off-target activity, the likelihood that they share the same off-target site (and that this off-target site has ACE2-reuglatory activity) should be extremely low. We have emphasized this aspect of the screen results in our revised Discussion (page 13).

**Reviewer #2:**

**In their revised manuscript, “Identification of cell type specific ACE2 modifiers by CRISPR screening” Sherman et al. expand their efforts to elucidate the regulatory pathways that control the expression of ACE2 in human cell lines. The updated submission incorporates several new experiments, most in direct response to the prior reviews. Perhaps the most significant issue in the initial submission, noted by both reviewers, was the investigation of a single cell line (Huh7) for the identification of factors that modulate ACE2 expression (with notably heterogeneous expression patterns), which raised concerns about the generalizability of the findings. In response, the authors now report additional focused CRISPR screens for ACE2-modulating genes in Calu-3 cells. These screens, and the complementary validation experiments that accompany them, are well-designed, conducted with appropriate controls, and yield robust, biologically-interesting results. The Calu-3 lung adenocarcinoma cell line is a good choice for these studies and has been a workhorse cell line for multiple SARS-CoV-2 studies. The authors demonstrate that ACE2 expression patterns in Calu-3 are similarly heterogeneous to Huh7. However, remarkably, the genes identified as modulating ACE2 expression in Calu-3 cells were distinct from those identified in Huh7 cells. Thus, at least for the two cell lines evaluated, the screens were not able to identify “central” and/or “common” regulators of ACE2. The authors note that this outcome highlights the cell type-specific nature of ACE2 expression regulation, which in turn could impact cell type-specific phenomena related to SARS-CoV-2 infection. These cell type-specific results could represent significant and biologically relevant findings; as such, they likely warrant comparative studies exploring the distinct ACE2 regulatory mechanisms at play in different cell types, which are absent from the revised manuscript. The authors might explore genetic, epigenetic, and/or signaling pathway differences in Calu-3 and Huh7, much of which could be assessed using publicly available datasets. For example, the apparent absence of SMAD4-mediated regulation of ACE2 expression in Calu-3 cells (as compared to Huh7 results) is intriguing, and the expression of SMAD4 pathway components could be measured. In summary, in responding to initial reviews, the authors added well-conducted experiments with intriguing results, that unexpectedly revealed cell-type specific ACE2 regulations. Some additional indication as to the underlying reason(s) for the discrepancy between the tested cell lines would dramatically increase impact and context of the study.**

We thank Reviewer #2 for this insightful summary of our revision, and for the suggested approach to dissect the molecular basis of cell type specificity for ACE2 regulation. We analyzed a publicly available RNA-seq database (Broad Institute DepMap public release version 21Q4, <https://doi.org/10.6084/m9.figshare.16924132.v1>) and did not find a major difference in *SMAD4* mRNA abundance between Calu-3 and HuH7 cells. This observation however does not address the possibility of differential SMAD4 posttranscriptional regulation in either cell line. It is also possible that even with equal SMAD4 activity, *ACE2* expression may be differentially responsive in either cell line due to the presence of tissue-specific *cis*-regulatory elements. Distinguishing among these possibilities will be a focus for our future investigations. We have expanded on our discussion of these potential explanations in our revised manuscript (page 17).
